# Deletion of CDKAL1 Affects High-Fat Diet–Induced Fat Accumulation and Glucose-Stimulated Insulin Secretion in Mice, Indicating Relevance to Diabetes

**DOI:** 10.1371/journal.pone.0049055

**Published:** 2012-11-16

**Authors:** Tadashi Okamura, Rieko Yanobu-Takanashi, Fumihiko Takeuchi, Masato Isono, Koichi Akiyama, Yukiko Shimizu, Motohito Goto, Yi-Qiang Liang, Ken Yamamoto, Tomohiro Katsuya, Akihiro Fujioka, Keizo Ohnaka, Ryoichi Takayanagi, Toshio Ogihara, Yukio Yamori, Norihiro Kato

**Affiliations:** 1 Division of Animal Model, Department of Infectious Diseases, Research Institute, National Center for Global Health and Medicine, Tokyo, Japan; 2 Department of Gene Diagnostics and Therapeutics, Research Institute, National Center for Global Health and Medicine, Tokyo, Japan; 3 Department of Molecular Genetics, Medical Institute of Bioregulation, Kyushu University, Fukuoka, Japan; 4 Department of Clinical Gene Therapy, Osaka University Graduate School of Medicine, Suita, Japan; 5 Department of Geriatric Medicine and Nephrology, Osaka University Graduate School of Medicine, Suita, Japan; 6 Amagasaki Health Medical Foundation, Amagasaki Japan; 7 Department of Geriatric Medicine, Graduate School of Medical Sciences, Kyushu University, Fukuoka, Japan; 8 Department of Medicine and Bioregulatory Science, Graduate School of Medical Sciences, Kyushu University, Fukuoka, Japan; 9 Morinomiya University of Medical Sciences, Osaka, Japan; 10 Mukogawa Women’s University Institute for World Health Development, Mukogawa, Japan; University of Bremen, Germany

## Abstract

**Background/Objective:**

The *CDKAL1* gene is among the best-replicated susceptibility loci for type 2 diabetes, originally identified by genome-wide association studies in humans. To clarify a physiological importance of CDKAL1, we examined effects of a global *Cdkal1*-null mutation in mice and also evaluated the influence of a *CDKAL1* risk allele on body mass index (BMI) in Japanese subjects.

**Methods:**

In *Cdkal1*-deficient (*Cdkal1* ^−/−^) mice, we performed oral glucose tolerance test, insulin tolerance test, and perfusion experiments with and without high-fat feeding. Based on the findings in mice, we tested genetic association of *CDKAL1* variants with BMI, as a measure of adiposity, and type 2 diabetes in Japanese.

**Principal Findings:**

On a standard diet, *Cdkal1* ^−/−^ mice were modestly lighter in weight than wild-type littermates without major alterations in glucose metabolism. On a high fat diet, *Cdkal1* ^−/−^ mice showed significant reduction in fat accumulation (17% reduction in %intraabdominal fat, *P* = 0.023 vs. wild-type littermates) with less impaired insulin sensitivity at an early stage. High fat feeding did not potentiate insulin secretion in *Cdkal1* ^−/−^ mice (1.0-fold), contrary to the results in wild-type littermates (1.6-fold, *P*<0.01). Inversely, at a later stage, *Cdkal1* ^−/−^ mice showed more prominent impairment of insulin sensitivity and glucose tolerance. mRNA expression analysis indicated that *Scd1* might function as a critical mediator of the altered metabolism in *Cdkal1* ^−/−^ mice. In accordance with the findings in mice, a nominally significant (*P*<0.05) association between *CDKAL1* rs4712523 and BMI was replicated in 2 Japanese general populations comprising 5,695 and 12,569 samples; the risk allele for type 2 diabetes was also associated with decreased BMI.

**Conclusions:**

*Cdkal1* gene deletion is accompanied by modestly impaired insulin secretion and longitudinal fluctuations in insulin sensitivity during high-fat feeding in mice. CDKAL1 may affect such compensatory mechanisms regulating glucose homeostasis through interaction with diet.

## Introduction

Genome-wide association (GWA) studies have facilitated the identification of genetic regions involved in the development of type 2 diabetes. Robust evidence of disease association in different populations has been obtained for several novel susceptibility gene loci identified by GWA studies; *CDKAL1* is among the best-replicated susceptibility loci [1]–[6]. The *CDKAL1* gene encodes a 65 kDa protein– cyclin-dependent kinase 5 regulatory subunit associated protein 1-like 1 (CDKAL1). A cluster of single nucleotide polymorphisms (SNPs) in intron 5 of the *CDKAL1* gene were associated with type 2 diabetes in populations of European and Asian descent [6]. This association was further tested with phenotypes of β-cell dysfunction, in particular, impaired insulin secretion as assessed by the oral or intravenous glucose tolerance test or a hyperglycemic clamp, showing reproducible association of the same variants with reduced first-phase insulin secretion [7]–[10]. In addition, it was reported that the type 2 diabetes risk–conferring alleles of *CDKAL1* were associated with lower birth weight [11], which is known to be associated with an increased risk of type 2 diabetes, presumably due to reduced insulin secretion or insulin sensitivity, i.e., the fetal insulin hypothesis [12]. Together, despite a lack of direct biological evidence reported to date, these association data support the possible contribution of causal variants at *CDKAL1* to the pathogenesis of type 2 diabetes.

In the present study, we first examined the effect of a global *Cdkal1*-null mutation in a mouse model to clarify the physiological importance of CDKAL1. Further, to see whether the observations of altered fat accumulation and reduced body weight in *Cdkal1*-deficient mice, not at birth but adulthood, could be pertinent to the human situation, we tested an association of *CDKAL1* with adult body mass index (BMI) in 2 independent Japanese populations and detected reproducible associations.

## Materials and Methods

### Ethics Statement

All animal experiments were approved by the Animal Care and Use Committee of the National Center for Global Health and Medicine (NCGM) Research Institute (permit number: 23-Tg-31), and conducted in accordance with institutional procedures. All human participants provided written informed consent, and the ethics committees of NCGM, Kyushu University, Osaka University, and Amagasaki Health Medical Foundation approved the protocols.

### Generation of *Cdkal1*-knockout Mice

**Figure 1:**
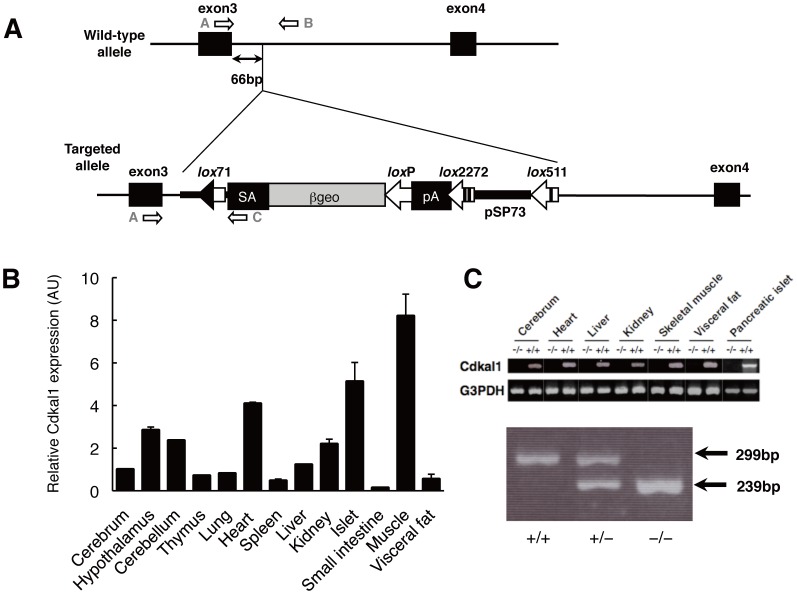
Generation of *Cdkal1*-knockout mice. **A:** Integration site of the trap vector. Filled boxes represent exons 3 and 4 of the *Cdkal1* gene. The trap vector was inserted 66-bp downstream from exon 3. The trap vector pU17 contains an intron and a splice acceptor (SA) sequence from the mouse *En-2* gene, the *ßgeo* gene, and polyadenylation signal (pA). A *lox*71 site is located within the intron sequences, and *lox*P, *lox*2272, and *lox*511 sites are located downstream of the *ßgeo,* pA, and pSP73 vector sequences, respectively. The arrows indicate the primers used for genotyping. The primer sequences were: (*A*) CGAAGGCGGAATACCCAAA, (*B*) TCATATCCGTTCCCCTAATTCCC, and (*C*) GTCCCCCTTCCTATGTAACCCAC. **B:** Relative mRNA expression of *Cdkal1* in a series of tissues/organs from C57BL/6 mice. Total RNA was isolated from various tissues of mice, treated with DNase, and then reverse-transcribed using ReverTra Ace (Toyobo, Osaka, Japan) and random primers. PCR was performed using a primer pair to specifically amplify part of the mouse *Cdkal1* gene (1090 bp in size), from exon 3 to 12∶5′-CGAAGGCGGAATACCCAAA-3′ and 5′-CAGCAGGAGTTCCGGGTCTT-3′. Quantitative reverse-transcription (RT) PCR was performed using a *Cdkal1-*specific primer and probe (Mm00507443_m1) (Applied Biosystems, Foster City, CA, USA). The values were arbitrary units after normalization with actin, beta (Actb). **C:** RT-PCR analysis of selected tissues of *Cdkal1* ^+/+^and *Cdkal1* ^−/−^ mice (*upper panel*). Gapdh mRNA was used as a positive control. PCR analysis for genotyping (*lower panel*). DNA fragments from the wild-type (299 bp) and target (239 bp) alleles were amplified using primer pairs *A*/*B* and *A*/*C*, respectively.

*Cdkal1*-knockout mice were generated by the gene trapping method [13], [14] at TransGenic Inc. (Kobe, Japan) and thereafter established as an experimental model at NCGM. An ES cell line TT2 [15], which had a mixed genetic background of CBA/JNCrj and C57BL/6J, was used for gene trapping. 5′-rapid amplification of cDNA ends (RACE) and sequence analysis showed that the gene trap vector pU17 was successfully inserted into intron 3 of the *Cdkal1* gene (Figure 1A). Germ-line transmitting chimaeric mice were generated and mated with C57BL/6 females. The F1 heterozygous mice (*Cdkal1* ^+/−^) were examined for the presence of the trap gene by PCR. The F1 heterozygous mice were backcrossed at least twice onto a C57BL/6 background and then interbred to generate *Cdkal1* ^−/−^ mice. Their wild-type littermates (*Cdkal1* ^+/+^) were used as the controls. All mice were housed in air-conditioned animal rooms at an ambient air temperature of 22±2°C and relative humidity of 50±15%, under specific pathogen-free conditions with a 12-h light/dark cycle. Male mice were used for the experiments; they were weaned at 4 weeks of age, given free access to drinking water, and were fed a standard diet (CE-2, 12 kcal%, CLEA Japan, Inc., Tokyo, Japan). Some mice were switched to a high-fat diet (D12451, 45 kcal%, Research diet, New Brunswick, NJ, USA) at 8 weeks of age (Table S1).

### Metabolic Studies

Blood samples were obtained after 15-h fast. Plasma glucose levels were measured with a Glucose C-II kit (Wako Pure Chemical Industries, Osaka, Japan). Plasma insulin concentrations were determined by an ultra-high sensitivity mouse insulin enzyme-linked immunosorbent assay kit (Morinaga, Yokohama, Japan). Total cholesterol (TC) and triglyceride (TG) levels in plasma, the liver and muscle were measured with a cholesterol C-II kit and a triglyceride E kit (Wako Pure Chemical Industries, Osaka, Japan), respectively. Insulin contents were measured in the whole pancreas according to the protocols described previously [16], [17].

### Assessment of Locomotor Activity

Locomotor activity was assessed by scoring the number of photobeam breaks in an open field test chamber (40 cm×40 cm×35 cm) (Panlab S. I., Barcelona, Spain). The 16-weeks-old male mice fed on a high-fat diet for 8 weeks were used for the open field test. Each mouse was placed in the chamber and habituated for 2 h and its spontaneous locomotor activities were recorded for the following 60 min.

### Oral Glucose Tolerance Test (OGTT) and Insulin Tolerance Test (ITT)

Glucose tolerance was assessed by oral glucose administration. After a 15-h fast, the mice were weighed and glucose (1 g/kg or 2 g/kg) was administered orally. Glucose and insulin concentrations in the blood of tail vein and retro-orbital venous plexus, respectively, were measured. For ITT, mice fasted for 3 h, and human insulin (1 IU/kg or 0.75 IU/kg; Novolin R, Novo Nordisk, Denmark) was administered via intraperitoneal injection. Blood samples were drawn from the tail vein at different time points. The glucose value at each time point was expressed as a percentage of the value at time 0.

### Perfusion Experiments in Mouse Pancreata

Overnight-fasted mice (*Cdkal1* ^+/+^and *Cdkal1* ^−/−^) at 12 weeks of age, which were fed either standard diet or high fat diet, were used in perfusion experiments as previously reported [18] with slight modifications. Briefly, after anesthesia, the superior mesenteric and renal arteries were ligated, and the aorta was tied off just below the diaphragm. The perfusate was infused from a catheter placed in the aorta and collected from the portal vein. The perfusion protocols began with a 20-min equilibration period with Krebs-Ringer bicarbonate (KRB) buffer containing 2.8 mmol/L glucose. At 3 min after sampling initiation, the glucose concentration of the perfusate was shifted from 2.8 mmol/L to 16.7 mmol/L during the subsequent period of 17 min. The flow rate of the perfusate was 1 ml/min. All samples were readily collected on ice and stored at −80°C. Insulin concentrations in the perfusate were measured as described above.

### CT Scan

Intraabdominal and subcutaneous fat of mice was examined radiographically using LaTheta LCT-200 (ALOKA, Mitaka, Japan) according to the manufacturer’s protocol. CT scanning was performed at 1.5-mm intervals from the diaphragm to the bottom of the abdominal cavity.

### Microarray Analysis

Microarray gene expression analysis was performed on the muscle from *Cdkal1* ^−/−^ mice and wild-type littermates (*n* = 4 each) using a mouse whole-genome microarray kit ver2.0 (Agilent Technologies, Palo Alto, CA, USA) according to the manufacturer’s protocol. The data analysis was done with the Bioconductor software (http://www.bioconductor.org/).

### Quantitative Real-time PCR

For the preparation of RNA, Isogen (Nippon Gene, Tokyo, Japan) was used. cDNA synthesis was performed with random hexamer-oligonucleotides. Quantitative PCR was performed on the 7900HT Fast Real-Time PCR system (Applied Biosystems, Foster City, CA, USA) with FastStart Universal SYBR Green Master (Roche Diagnostics, Mannheim, Germany). The primers used for the PCR amplification are shown in Table S2.

### Immunoblot Analysis

For western blotting, 5 units of human regular insulin was injected into the inferior vena cava of anesthetized mice after overnight fast, and the livers were removed at 2 min, the hind limb muscles and white adipose tissues (WAT) at 5 min after injection. The tissues were then homogenized in the lysis buffer and protease inhibitor cocktail (Roche Diagnostics, Mannheim, Germany). Fifty micrograms of protein was loaded to a 12% SDS/PAGE gel, and transferred onto PVDF membrane (BIO-Rad, Hercules CA, USA). We purchased total-Akt (#4691) and phospho-Akt (pAkt, #9271) antibodies (Cell Signaling, Beverly MA, USA) and Cdkal1 (#ab68045) antibody (Abcam, Cambridge MA, USA). The proteins were visualized by using ECL (Thermo Fisher Scientific, Waltham MA, USA) and quantified by densitometry.

### Human Study Sample

An association study of 7 previously identified variants for type 2 diabetes and/or BMI was performed in Japanese subjects (Text S1); 5,695 samples of the Amagasaki panel [19] and 12,569 samples of the Fukuoka panel [20]; a case-control study panel comprising 6,369 cases and 6,406 controls, where type 2 diabetes was diagnosed according to the 1999 WHO criteria as described elsewhere [5]. Brief descriptions of the assessment of biological parameters and lifestyle are available elsewhere [19], [20].

### SNP Genotyping

Samples were genotyped using the TaqMan assay (Life Technologies Japan, Tokyo, Japan) for 7 SNPs from 7 unique loci. These included *CDKAL1* (rs4712523), *IGF2BP2* (rs4402960), *SLC30A8* (rs13266634), *CDKN2A/B* (rs2383208), *HHEX* (rs1111875), *TCF7L2* (rs7903146), and *KCNQ1* (rs2237892). The genotype distribution of all tested SNPs was in Hardy-Weinberg equilibrium (*P*>0.01). We obtained successful genotyping call rates of>99% for the whole characterized sample.

### Statistical Analysis

#### Comparative analysis in mice

The results are expressed as means ± SEM unless otherwise indicated. Differences were analyzed using an unpaired Student’s *t*-test when comparing two groups means. Data for body weight and insulin secretion during OGTT were subjected to repeated measure ANOVA. *P*<0.05 was considered to be statistically significant.

#### SNP association analysis

We standardized BMI to the *z*-score in each panel before association analysis. SNPs were tested for association with BMI using linear regression analysis in the additive genotype model after adjustment for age classes separately by sex. Age classes were defined according to the age distribution in the individual panels; they were ≤40, 41–50, 51–60, and>60 (Amagasaki panel); and ≤55, 56–60, 61–65, 66–70, and>70 (Fukuoka panel). Association results for the two Japanese panels were combined using the inverse variance method. We used PLINK (http://pngu.mgh.harvard.edu/~purcell/plink/) [21], R software (version 2.8.1; www.r-project.org), and the rmeta package (http://cran.r-project.org) for association tests and meta-analysis. A significance level was set at *P*<0.007 according to Bonferroni correction for multiple testing.

## Results

### Phenotypic Characterization of *Cdkal1*-knockout Mice

Expression of *Cdkal1* was found to be relatively ubiquitous in mice. *Cdkal1* mRNA was prominently expressed in the skeletal muscle, pancreatic islets, and heart (Figures 1B and 1C), as previously reported in humans [22]. Here, CDKAL1 protein was confirmed to be detectable in *Cdkal1* ^+/+^but not in *Cdkal1* ^−/−^ mice (Figure S1C) as previously reported [23]. Genotype analysis of 91 pups generated by cross-breeding heterozygous *Cdkal1* ^+/−^ mice demonstrated that 21 mice were *Cdkal1* ^+/+^, 46 were *Cdkal1* ^+/−^ and 24 were *Cdkal1* ^−/−^, a distribution consistent with Mendelian inheritance. The locomotor activities of *Cdkal1* ^−/−^ mice were indistinguishable from their wild-type littermates (Figure S2) and no gross anatomical changes were observed either externally or in the major organs.

**Figure 2:**
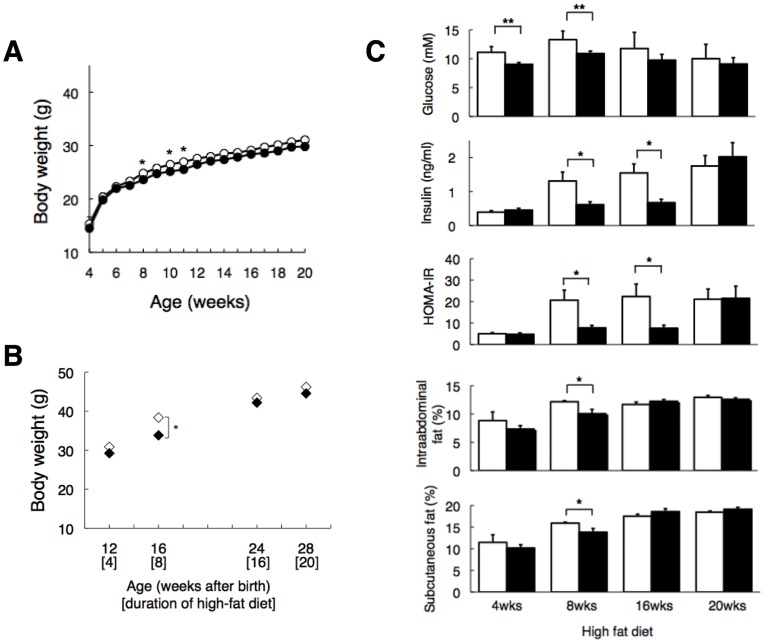
Phenotype comparison between wild-type littermates (WT, *Cdkal1^+/+^*) and *Cdkal1* knockout (KO, *Cdkal1* ^−/−^) mice. **A:** Weight curves from animals on a standard diet [WT (*n* = 11) vs. KO (*n* = 12)]. *P* = 0.15; F (1, 16) = 2.3 by repeated measure ANOVA. **B:** Weight was measured after 4, 8, 16, and 20 weeks on a high fat diet [WT (*n* = 21–26) vs. KO (*n* = 19–25)]. *P* = 0.18; F (1, 3) = 1.9 by repeated measure ANOVA. **C:** Plasma glucose (mM) and insulin (ng/ml) were measured; these were used to calculate HOMA-IR [HOMA-IR = G × I (in ng/ml) × 1.16 = G × I (in µIU/ml)/22.5]. %intraabdominal and %subcutaneous fat was calculated by dividing intraabdominal and subcutaneous fat content, measured using CT scan, with body weight. *P<0.05, **P<0.01.

Table 1 summarizes the phenotypes in these animals. On a standard diet, although the effects on body weight were relatively modest (i.e., reduction by 2–5%) in *Cdkal1* ^−/−^ mice compared to wild-type littermates (see Figure 2A), we observed significant, symmetrical increases of body weight in *Cdkal1* transgenic mice, which we further generated (Text S1 and Figure S1). This supported the possible involvement of Cdkal1 in the regulation of body weight. No significant changes in plasma lipids, glucose, or insulin were observed in *Cdkal1* ^−/−^ mice relative to wild-type littermates. On a high-fat (high-saturated fat/low-carbohydrate) diet, *Cdkal1* ^−/−^ mice also tended to be lighter throughout the follow-up period. In particular, *Cdkal1* ^−/−^ mice showed a significant reduction in fat accumulation, monitored by %intraabdominal and %subcutaneous fat, after 8 weeks on the high-fat diet, i.e., 16 weeks of age (see Figures 2B and 2C). These findings were further supported by reduced lipid accumulation (as estimated by triglyceride content) in the liver and muscle at the corresponding time point (Figure S3). Inversely, at a later stage (20 weeks) of high-fat feeding, lipid accumulation became more prominent in *Cdkal1* ^−/−^ mice than wild-type littermates (Figure S3). Food intake tended to be reduced in *Cdkal1* ^−/−^mice (Table 1).

**Table 1:** Phenotypic characterization of Cdkal1-knockout mice with and without high-fat feeding.

Variables		Wild-type (WT)	*Cdcal1* Knockout (KO)	*P-value*, *t*-test (WT vs. KO)
*Body composition*				
	Body weight (g)			
	STD	31.34±0.48 (11)	29.22±1.10 (12)	0.095
	4wks of HFD	30.85±0.77 (21)	29.22±0.57 (20)	0.098
	8wks of HFD	38.36±0.88 (26)	33.81±0.78 (25)	3.4×10^−4^
	16wks of HFD	43.40±1.19 (23)	42.19±1.18 (21)	0.473
	20wks of HFD	46.20±1.06 (21)	44.55±0.96 (19)	0.256
	Visceral fat per body weight (%)			
	STD	5.38±0.47 (11)	4.92±0.89 (12)	0.659
	4wks of HFD	8.84±1.53 (5)	7.34±0.60 (5)	0.389
	8wks of HFD	12.17±0.19 (10)	10.06±0.76 (10)	0.023
	16wks of HFD	11.69±0.42 (8)	12.25±0.31 (7)	0.320
	20wks of HFD	12.95±0.33 (16)	12.60±0.27 (14)	0.421
*Food intake*				
	Food intake (g/day)			
	STD	3.89±0.08 (4)	3.59±0.09 (4)	0.049
	4wks of HFD	2.95±0.06 (31)	2.86±0.05 (17)	0.320
	8wks of HFD	2.88±0.05 (20)	2.57±0.07 (18)	5.2×10^−4^
	16wks of HFD	2.77±0.06 (16)	2.56±0.04 (15)	0.008
	20wks of HFD	2.82±0.06 (16)	2.73±0.06 (14)	0.284
	Food intake per body weight (g/day)			
	STD	0.117±0.005 (4)	0.114±0.006 (4)	0.759
	4wks of HFD	0.093±0.002 (31)	0.099±0.002 (17)	0.054
	8wks of HFD	0.073±0.002 (20)	0.076±0.002 (18)	0.156
	16wks of HFD	0.060±0.002 (16)	0.057±0.001 (15)	0.129
	20wks of HFD	0.059±0.001 (16)	0.059±0.001 (14)	0.928
*Insulin (ng/ml)*				
	Fasting plasma insulin			
	STD	0.50±0.06 (10)	0.57±0.08 (9)	0.493
	4wks of HFD	0.39±0.04 (11)	0.45±0.05 (9)	0.361
	8wks of HFD	1.31±0.26 (9)	0.61±0.08 (9)	0.031
	16wks of HFD	1.55±0.27 (5)	0.67±0.10 (5)	0.015
	20wks of HFD	1.75±0.31 (5)	2.02±0.41 (5)	0.612
	Pancreas insulin content (ng) per pancreas weight (mg)			
	STD	104.8±13.1 (6)	117.9±13.1 (6)	0.451
	8wks of HFD	107.7±10.5 (6)	134.9±19.8 (6)	0.252
	16wks of HFD	138.8±21.6 (4)	101.2±15.4 (4)	0.204
*Leptin (ng/ml)*				
	20wks of HFD	28.1±1.1 (12)	31.2±1.2 (10)	0.078
*TNFα (pg/ml)*				
	20wks of HFD	33.7±4.8 (13)	26.6±4.0 (11)	0.281
*Serum lipids*				
	Triacylglycerol (mg/dl)			
	STD	62.9±17.1 (4)	60.0±15.8 (6)	0.906
	8wks of HFD	58.0±6.1 (10)	64.7±4.7 (10)	0.399
	16wks of HFD	65.0±8.2 (7)	63.7±5.9 (5)	0.906
	20wks of HFD	65.2±3.2 (14)	70.1±4.2 (9)	0.359
	Total cholesterol (mg/dl)			
	STD	85.8±13.4 (4)	65.7±3.2 (6)	0.231
	8wks of HFD	164.4±7.5 (10)	137.3±2.7 (10)	0.005
	16wks of HFD	107.6±13.2 (7)	127.8±8.6 (5)	0.270
	20wks of HFD	169.3±6.0 (8)	162.2±11.2 (9)	0.593
	Free fatty acid (mEq/l)			
	STD	0.90±0.1 (4)	1.09±0.2 (6)	0.375
	8wks of HFD	1.03±0.1 (10)	1.10±0.1 (10)	0.689
	16wks of HFD	1.20±0.2 (7)	1.59±0.2 (5)	0.191
	20wks of HFD	0.96±0.03 (8)	1.10±0.06 (9)	0.051

Values are means ± SEM. The number of animals (all males) in each group is shown in parentheses.

Mice were weaned at 4weeks (wks) of age. For the standard diet (STD) group, all mice were characterized at 30 wks of age. For the high fat diet (HFD) group, the diet was shifted from STD to HFD at 8 wks of age; i.e., mice in the 4 wks of HFD group were 12 wks of age.

Plasma levels of insulin, glucose, leptin, TNFa, and lipids were measured after 16-hr fast.

During the follow-up period, *Cdkal1* ^−/−^mice caught up with wild-type littermates in %intraabdominal fat after 16 weeks on the high-fat diet (Figure 2C). Plasma glucose and insulin levels remained lower in *Cdkal1* ^−/−^mice over almost the entire follow-up period, except that plasma insulin eventually increased in *Cdkal1* ^−/−^mice after 20 weeks on the high-fat diet. At this time point, despite almost equivalent levels of insulin, and %intraabdominal and %subcutaneous fat between the strains, plasma leptin levels tended to be higher in *Cdkal1* ^−/−^mice than wild-type littermates (28.1±1.1 ng/ml vs. 31.2±1.2 ng/ml for wild-type littermates vs. *Cdkal1* ^−/−^ mice, *P* = 0.078). They did not accompany the expected increases in TNF-α (Table 1), whose circulatory levels are known to generally increase with the degree of obesity [24]. On a high-fat diet, *Cdkal1* ^−/−^mice were more insulin-sensitive than wild-type littermates until a certain time point; that is, a lower glucose level was attainable with a significantly (*P* = 0.015) lower insulin level despite almost equivalent fat accumulation after 16 weeks on the high-fat diet (Figure 2C).

### Glucose Homeostasis and Insulin Release *in vivo*

**Figure 3:**
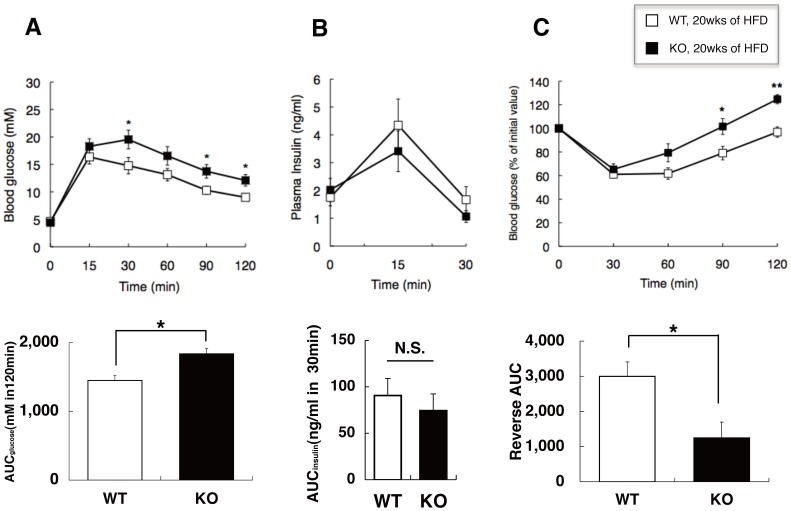
Glucose tolerance (A, B) and insulin tolerance tests (C) in WT and *Cdkal1* KO mice. In mice after 20 weeks on a high fat diet (HFD), (**A**) oral glucose tolerance was assessed by OGTT [1 g/kg glucose, WT (*n* = 12) vs. KO (*n* = 10)]; (**B**) insulin levels were measured during OGTT [WT (*n* = 5) vs. KO (*n* = 5)]; and (**C**) insulin sensitivity was assessed by ITT [IU/kg, WT (*n* = 12) vs. KO (*n* = 11)]. In OGTT and ITT, glucose levels were measured in whole blood from tail vein with GluTest blood glucose monitor (Sanwa Kagaku, Nagoya, Japan), while insulin levels were determined in plasma with an ultrahigh-sensitivity mouse insulin ELISA kit (Morinaga, Yokohama, Japan). In the bottom row, significant (*P*<0.05) inter-strain differences were found for (**A**) AUC_glucose_ and (**C**) AUC_insulin_ (see also Figure S4). *P<0.05, **P<0.01 compared with wild-type littermates; N.S., not significant. AUC, the areas under the curve.

On a standard diet, no apparent impairment of glucose tolerance was observed in *Cdkal1* ^−/−^mice at 12 and 20 weeks of age (Figure S4A and S4B). While a modest (but not significant) decrease in plasma glucose was seen at 60–120 min after insulin injection at both ages in *Cdkal1* ^−/−^mice relative to wild-type littermates, insulin sensitivity was almost indistinguishable between the two strains on a standard diet (Figure S4C and S4D). On a high-fat diet, elevated fasting glucose levels and glucose intolerance during OGTT were seen in both strains at an early stage (after 4 and 8 weeks) of high-fat feeding, which was more prominent in wild-type littermates than *Cdkal1* ^−/−^ mice (Figure S4E and S4F). Also, in wild-type littermates on a high-fat diet, insulin sensitivity assessed by ITT deteriorated more markedly (Figure S4G and S4H). However, at a later stage (after 20 weeks) of high-fat feeding (Figure 3), the impairment of glucose tolerance and insulin sensitivity became more prominent in *Cdkal1* ^−/−^ mice than wild-type littermates (*P*<0.05; Figures 3A and 3C), contrary to the findings at the early stage. Insulin secretion during OGTT was reduced in *Cdkal1* ^−/−^ mice on a high-fat diet (in particular, at an early stage of high-fat feeding) but not on a standard diet (Figure 3B and Figure S4I and S4J).

### Insulin Secretion in Perfused Pancreata

**Figure 4:**
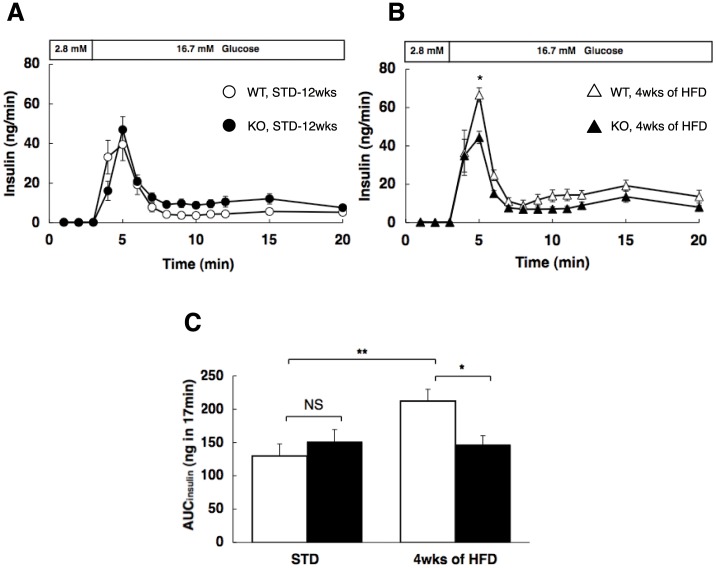
Insulin secretion in perfused pancreata of WT and *Cdkal1* KO mice. Mice were fed on a standard diet (WT, *n* = 6; KO, *n* = 8) (**A**) and a high fat diet (WT, *n* = 6; KO, *n* = 6) (**B**), in response to high glucose. Glucose concentration was shifted from 2.8 mM to 16.7 mM at 3-min. (**C**) The amounts of secreted insulin of WT (open bar) and KO (solid bar) mice after glucose stimulation are expressed as the AUC_insulin_ from 3 to 20-min. The areas under the curve were assessed for insulin levels in perfusate (AUC_insulin_) with the trapezoidal rule of suprabasal values. *P<0.05, **P<0.01.

To examine the time course of the insulin secretory response to high glucose in *Cdkal1* ^−/−^ mice, perfusion experiments were performed in the standard (Figure 4A) and high-fat (Figure 4B) diet groups. In wild-type littermates (*Cdkal1* ^+/+^), 16.7 mmol/L glucose elicited insulin secretion [the amount of secreted insulin (AUC_insulin_) after glucose stimulation (from 3 to 20 min); 130±18 ng in 17 min, *n* = 6], which was further potentiated by 4 weeks of high-fat feeding [AUC_insulin_; 212±18 ng, *n* = 6, *P*<0.01 vs. standard diet] (Figure 4C). In *Cdkal1* ^−/−^ mice, 16.7 mmol/L glucose elicited insulin secretion almost equivalently to *Cdkal1* ^+/+^mice on a standard diet (AUC_insulin_; 151±19 ng, *n* = 8), whereas there was no apparent potentiation of insulin secretion after 4 weeks of high-fat feeding (AUC_insulin_; 147±14 ng, *n* = 6). Thus, based on an assessment of AUC_insulin_, high-fat feeding did not potentiate insulin secretion in *Cdkal1* ^−/−^ mice (1.0-fold), contrary to the findings in *Cdkal1* ^+/+^mice (1.6-fold), although glucose tolerance was impaired in both strains as compared to the situation on a standard diet (Figure 4C and Figure S4A and S4E). It remains to be determined whether such inter-strain differences in impaired insulin secretion are reproducible in mice high-fat fed for 20 weeks, at which time points insulin sensitivity in *Cdkal1* ^−/−^ mice became worse and almost equivalent to that in *Cdkal1* ^+/+^mice (Figure 2C). Nevertheless, the present findings in perfusion experiments after 4 weeks of high-fat diet appeared to be consistent with the results for *in vitro* experiments (using batch incubated β cells) in the previous study [23].

### Changes Associated with Protection Against Diet-induced Obesity and Insulin Resistance in *Cdkal1* ^−/−^ Mice

**Figure 5:**
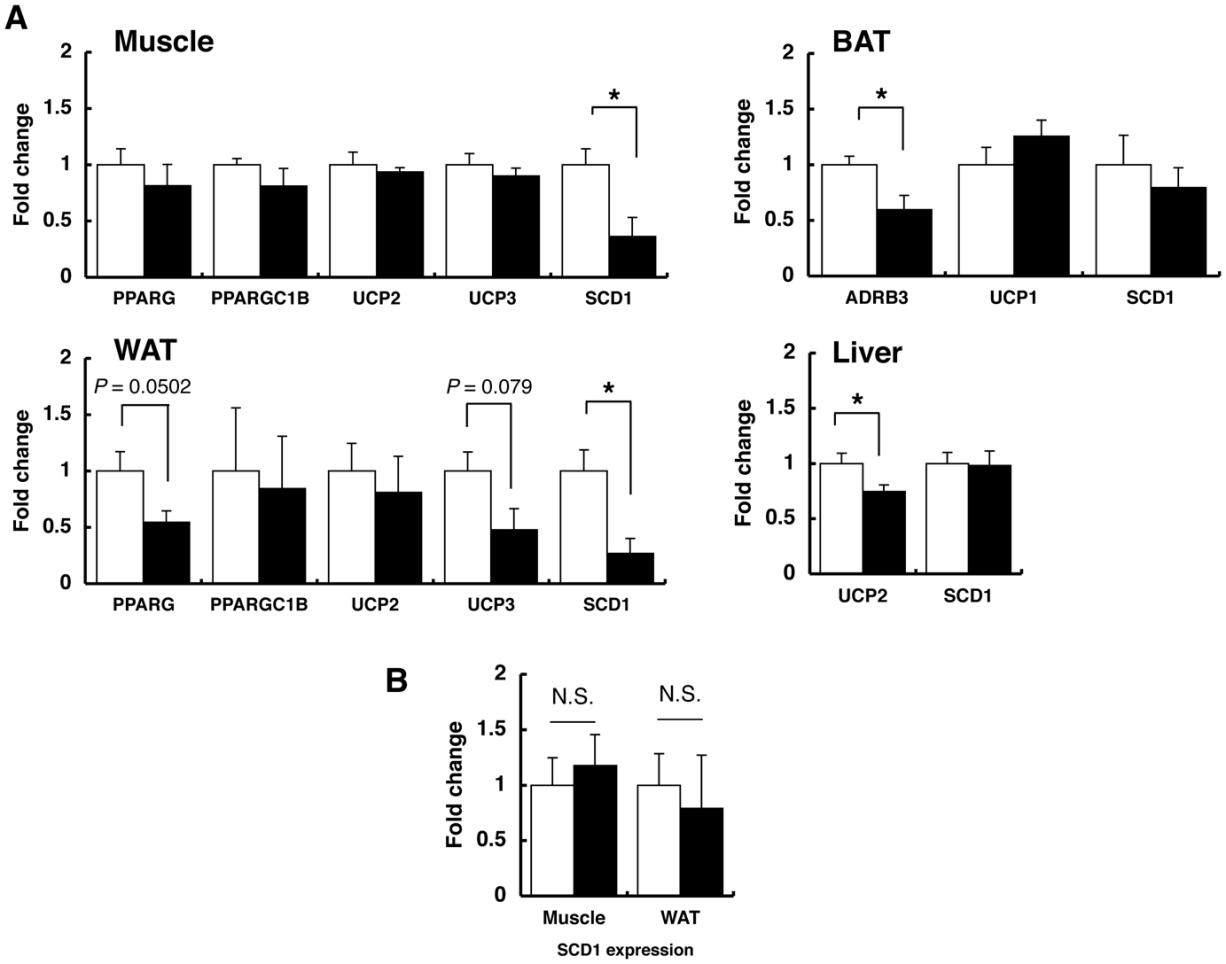
Reduced expression of *Scd1* in *Cdkal1*−/− muscle and white adipose tissue at the early stage of high fat diet. A: Quantification of mRNA for indicated genes in skeletal muscle, white (WAT) and brown (BAT) adipose tissues, and liver of WT (open bar, *n* = 4) and KO mice (solid bar, *n* = 4) fed on a high-fat diet for 10 weeks. **B:** Quantification of *Scd1* mRNA in the skeletal muscle and WAT of WT (open bar, *n* = 8) and KO mice (solid bar, *n* = 5) fed on a high-fat diet for 20 weeks. The mRNA expression of indicated genes was normalized to that of β-actin. The normalized data for KO mice are expressed relative to those for WT littermates. *P<0.05; N.S., not significant.

To understand biological mechanisms, by which *Cdkal1* ^−/−^ mice could show protection against high fat diet-induced obesity and insulin resistance, we performed microarray gene expression analysis on the muscle, along with comparing mRNA expression of target genes (including several physiological candidate genes) in brown adipose tissues (BAT) and WAT, liver and muscle between *Cdkal1* ^+/+^and *Cdkal1* ^−/−^ mice (Figure 5). Although there were significant (*P*<0.05) inter-strain differences in mRNA expression of a few candidate genes, e.g., *Adrb3* in BAT and *Ucp2* in the liver, they did not appear to be causal, considering the pattern of expression differences. In the microarray analysis, we identified an *Scd1* gene among a list of most significant genes differentially expressed (Table S3). *Scd1* was confirmed to be down-regulated in muscle and WAT of *Cdkal1* ^−/−^ mice (Figure 5).

Next, to test whether protection against high fat diet-induced insulin resistance is mediated by enhanced insulin signaling, we examined insulin-dependent Akt activation. We found that the levels of phosphorylated Akt protein were comparable between the strains when fed on a high-fat diet for 4 weeks (Figure S5).

### Correlation of type 2 Diabetes and BMI Associations at Candidate Loci

**Figure 6:**
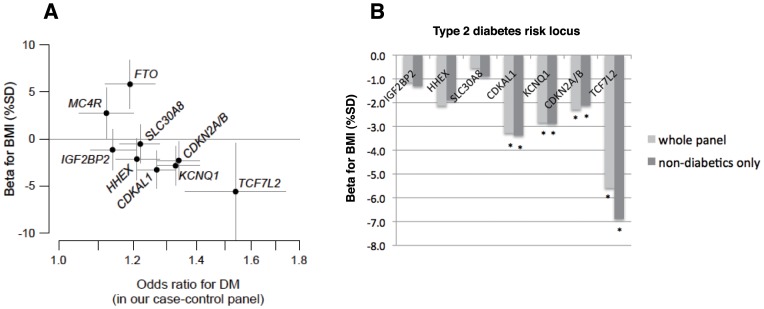
Effect size for type 2 diabetes risk and BMI at previously-reported candidate loci. The SNPs were previously reported to associate with type 2 diabetes and/or obesity. **A:** Genetic impacts on type 2 diabetes risk (OR in *x*-axis) and BMI level (β in *y*-axis) are compared for the SNPs. SNP rs numbers of individual loci are as follows: rs4402960 for *IGF2BP2*, rs4712523 for *CDKAL1*, rs13266634 for *SLC30A8*, rs2383208 for *CDKN2A/B*, rs1111875 for *HHEX*, rs7903146 for *TCF7L2*, and rs2237892 for *KCNQ1*. Data for *FTO* (rs9939609) and *MC4R* (rs12970134) were drawn from the previous report [29] for the purpose of comparison. Whiskers represent 95% CI. **B:** β of the individual SNP loci, which were found to negatively associate with BMI, when calculated in the whole population panel (light grey) and restricted to non-diabetic individuals (dark grey). *P<0.05.

In the Japanese general population samples (Table 2), we found significant (*P*<0.05/7 ≈ 0.007) associations with BMI at *CDKAL1* rs4712523 and *KCNQ1* rs2237892, and nominal (*P*<0.05) associations at 2 other loci–*CDKN2A/B* rs2383208 and *TCF7L2* rs7903146 (Table 3). At *CDKAL1*, we evaluated the association of rs4712523, which showed the strongest association in our previous GWA study of type 2 diabetes [5], with BMI and found reproducible evidence for association in 2 independent panels (Table 3), where the risk allele (G of rs4712523) for type 2 diabetes showed nominal association with lower BMI [*P* = 0.024 in the Amagasaki panel (*n* = 5,695) and *P* = 0.02 in the Fukuoka panel (*n* = 12,569)]. Even when restricted to non-diabetic individuals, the effect sizes were almost unchanged as compared to those calculated in the whole panel (Table S4). When we reanalyzed the BMI association at *CDKAL1* by arbitrarily categorizing the samples into two age groups (age<60 years and age≥60 years) without adjusting for age in the linear regression, there was no statistically significant inter-age-group difference (*P*_heterogeneity_ = 0.56, Table 3). In addition, the “inverse” correlation between disease risk and lower BMI was replicated for 6 other type 2 diabetes risk loci, which had been all suggested to affect insulin secretion (Figure 6). There was, on the other hand, a positive correlation between type 2 diabetes risk and higher BMI for 2 obesity (or insulin resistance)-associated loci–*FTO* and *MC4R*–as previously reported [25]–[29].

**Table 2:** Clinical characteristics of study participants.

Variables	Amagasaki panel		Fukuoka panel	
	Total	Non-diabetics only	Total	Non-diabetics only
Number of subjects [female/male]	5,695 [2,290/3,405]	5,349 [2,227/3,122]	12,569 [6,898/5,671]	11,307 [6,475/4,832]
Age (yr)	48.8 (12.6)	48.3 (12.6)	62.6 (6.8)	62.4 (6.8)
Body mass index (kg/m^2^)	23.0 (3.2)	22.9 (3.2)	23.1 (3.0)	23.0 (3.0)
Body weight (kg)	61.8 (11.5)	61.5 (11.4)	58.4 (10.2)	58.0 (10.1)
Alcohol drinking^a^				
None (%)	24.1	24.5	48.6	49.3
Previous drinker (%)	1.2	1.0	5.0	4.4
Chance drinker (%)	35.6	35.9	–	–
Current drinker (%)	39.1	38.6	46.4	46.3
Smoking				
None (%)	55.2	55.7	59.9	61.6
Previous smoker (%)	9.9	9.7	23.1	21.9
Current smoker (%)	34.8	34.6	17.0	16.5
Blood chemistry				
Fasting plasma glucose (mmol/l)	5.22 (0.54)	5.21 (0.54)	N/A	N/A
HbA1c^b^	5.41 (0.84)	5.22 (0.40)	5.23 (0.77)	5.05 (0.37)
LDL cholesterol (mg/dl)^c^	123.9 (31.2)	123.5 (31.0)	N/A	N/A
Triglycerides (mg/dl)	110.1 (85.9)	108.2 (83.9)	146.6 (99.3)	143.3 (93.7)
HDL cholesterol (mg/dl)	62.8 (17.7)	63.2 (17.8)	62.5 (16.8)	63.1 (16.8)

Values are means (SD) unless otherwise indicated.

All clinical assessments were performed using uniform standards in each population.

Blood samples were taken after ≥6 hours fast in the Amagasaki panel; without setting strict fasting condition in the Fukuoka panel.

a Since the questionnaire did not differeniate the category of chance drinker from that of current drinker, the corresponding subjects are combined for the category of current drinker in the Fukuoka panel.

b HbA1c was measured for 1,288 subjects in the Amagasaki panel; for all participants in the Fukuoka panel.

c LDL cholesterol was calculated in the Amagasaki panel using the Friedewald formula, with missing values assigned to individuals with triglycerides >400 mg/dl. Since blood samples were taken without setting strict fasting condition, the values of LDL cholesterol and the prevalence of dyslipidemia are not shown for the Fukuoka panel.

d Hypertension is defined when systolic blood pressure ≥140 mmHg and/or diastolic blood pressure ≥90 mmHg, or taking antihypertensive medication. Diabetes is defined when fasting plasma glucose ≥7.0 mmol/l and/or HbA1c ≥6.5%, or taking blood glucose lowering medication. Dyslipidemia is defined according to the Japan Atherosclerosis Society Guidelines (Teramoto et al. J Atheroscler Thromb 14∶155–158, 2007).

**Table 3:** Cohort-wise BMI association of SNPs genotyped in two general Japanese populations.

					Amagasaki (*n* = 5,695)			Fukuoka (*n* = 12,569)			Combined (*n* = 18,264)		
Chromo-some	Neighbouring gene	SNP rs #	Position (B37)	Allele tested	Tested allele freq.	BMI		Tested allele freq.	BMI		Tested allele freq.	BMI	
						BETA (%)	*P*		BETA (%)	*P*		BETA (%)	*P*
3	*IGF2BP2*	rs4402960	18,55,11,687	T	0.32	0.75	0.705	0.32	−2.00	0.133	0.32	−1.15	0.300
6	*CDKAL1*	rs4712523	2,06,57,564	G	0.43	−4.12	0.024	0.43	−2.88	0.020	0.43	−3.28	0.001
8	*SLC30A8*	rs13266634	11,81,84,783	C	0.59	1.42	0.442	0.58	−1.46	0.249	0.59	−0.54	0.605
9	*CDKN2A/B*	rs2383208	2,21,32,076	A	0.57	−2.96	0.108	0.57	−1.97	0.118	0.57	−2.29	0.028
10	*HHEX*	rs1111875	9,44,62,882	C	0.29	−1.11	0.573	0.28	−2.64	0.057	0.29	−2.13	0.060
10	*TCF7L2*	rs7903146	11,47,58,349	T	0.04	−6.81	0.149	0.04	−5.03	0.113	0.04	−5.59	0.034
11	*KCNQ1*	rs2237892	28,39,751	C	0.62	−1.23	0.509	0.61	−3.58	0.005	0.61	−2.84	0.007

Effect sizes are indicated as beta per SD unit of phenotype.Two-tailed P values are shown in the table.

T2D-risk, fasting plasma glucose (FPG)-increasing and BMI-increasing alleles reported in the previous studies are tested.

When we arbitrarily categorized the samples into two age groups (age<60 years and age≥60 years), there was no significant inter-age-group difference in BMI association at *CDKAL1* rs4712523; P = 0.076, β = −2.66 for age<60 years; P = 0.006, β = −3.85 for age≥60 years in the combined samples.

Part of the samples (414 individuals in the Amagasaki Study panel) were included in the GWA meta-analysis of BMI (ref.35,36), where proxy SNPs (*r^2^*>0.78 in HapMap JPT+CHB) were tested for SNP-BMI acid association at *CDKAL1*. For the purpose of comprehensive evaluation, all the Amagasaki Study samples consecutively-enrolled are included in the present study.

### Meta-analysis of Effect Size for type 2 Diabetes at the *CDKAL1* Locus

**Figure 7:**
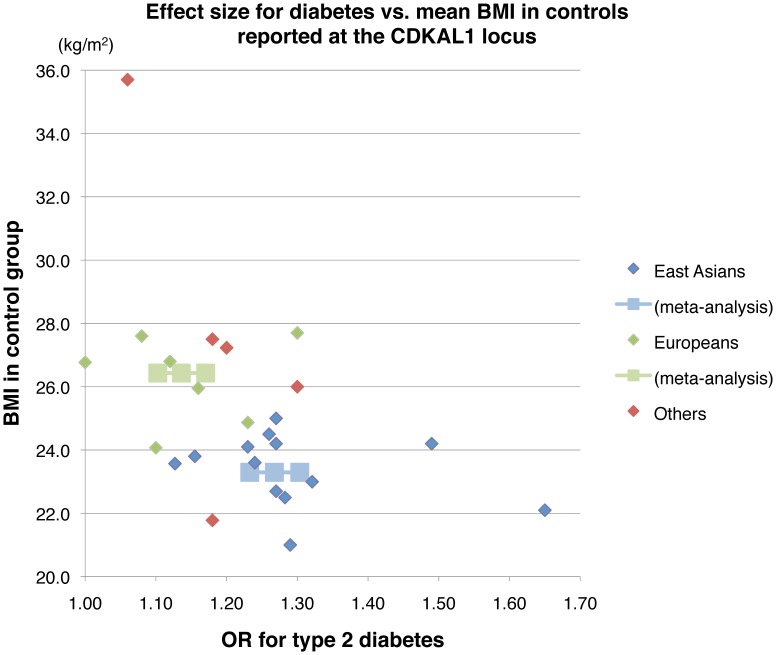
Relation between effect sizes for type 2 diabetes and BMI at *CDKAL1*. Meta-analysis was performed with the data in the control group (as the population mean) for reported case-control studies that had investigated the disease association at *CDKAL1.* The results for meta-analysis in East Asians (light blue) and Europeans (light green) are shown in the Figure. See Table S5 about the details of meta-analysis.

In the longitudinal follow-up, *Cdkal1* ^−/−^ mice gained adipose tissues and showed the deterioration of insulin sensitivity after a certain period of extreme high-fat feeding (Figure 2C). Analogously in the human, as dietary habits substantially influence BMI in the population at large [30], genetic effects on type 2 diabetes, attributable to *CDKAL1*, may differ between populations (or ethnic groups) with distinct dietary habits and the resultant cross-population diversity in BMI. To evaluate this possibility, we plotted effect sizes for type 2 diabetes against BMI in the control group (as the population mean data) for the individual case-control studies that investigated disease association at *CDKAL1* (Figure 7). There was a tendency toward inverse correlation between the 2 tested variables. As a consequence, we identified significant cross-population heterogeneity in effect sizes (*I^2^* = 96.4%, *P*<1.4×10^−7^) between East Asians (a relatively *thin* population, mean BMI = 23.3 kg/m^2^; OR = 1.27, 95% CI 1.23–1.30) and Europeans (a relatively *stout* population, mean BMI = 26.4 kg/m^2^; OR = 1.14, 95% CI 1.10–1.17) at *CDKAL1* (Table S5).

## Discussion

In *Cdkal1*-deficient mice, we tested the hypothesis that changes in the activity of Cdkal1 result in islet dysfunction and/or insulin resistance, thereby contributing to the pathogenesis of type 2 diabetes. Our results indicate that deletion of the *Cdkal1* gene results in a rather mild metabolic phenotype except a modest decrease in weight (Figure 2A) and a tendency toward enhanced insulin sensitivity (Figure S4C and S4D) on a standard diet. Of note is the fact that, whereas *Cdkal1*-deficient mice gain less fat (Figure 2C and Figure S3) and have less impaired glucose tolerance and insulin sensitivity (Figure 2C and Figure S4G and S4H) during the first 8 weeks of high-fat diet, their glucose tolerance and insulin sensitivity deteriorate after 20 weeks of fat enriched-diet compared to wild-type littermates (Figures 2C and 3A-3C). Together with the independent observations in human association studies, we speculate that *Cdkal1* modulates whole-body glucose metabolism in a bidirectional manner; that is, a lack of *Cdkal1* enhances insulin sensitivity presumably in the skeletal muscle, adipose tissue and/or liver, and impairs insulin secretion in the pancreatic islets. These compensatory mechanisms become evident with dietary intervention and may protect the mice against glucose intolerance or overt diabetes in the first place. However, after a certain period of extreme high-fat feeding, *Cdkal1* ^−/−^ mice come to gain fat, to show deterioration of insulin sensitivity, and eventually to exhibit apparent glucose intolerance.

Molecular variants that linked the human *CDKAL1* gene to increased type 2 diabetes susceptibility are located in the middle of a large intron of CDKAL1 [1]–[5]. It remains unknown whether the causal variant(s) can activate or attenuate the function of CDKAL1. Thus far, several human studies have indicated that the risk variant of *CDKAL1* is associated with reduced insulin secretion [7]–[10]. Besides the circumstantial evidence for reduced insulin secretion after a certain period of high-fat feeding in *Cdkal1* ^−/−^ mice (Figures 4B and 4C), two lines of evidence have supported our hypothesis that deletion of *Cdkal1* in mice impairs glucose tolerance and/or insulin secretion. First, by using β cells derived from *Cdkal1* ^−/−^ mice, we have verified that the number of fusion events during the first-phase insulin release is reduced *in vitro*, thereby leading to impairment of insulin exocytosis in *Cdkal1* ^−/−^ mice [23]. Also, it has been recently reported that pancreatic β cell-specific knockout mice show a decrease in insulin secretion and impaired blood glucose control, resulting from a reduction of glucose-stimulated proinsulin synthesis [31].

The most notable characteristic of *Cdkal1* ^−/−^ mice is the reduced fat accumulation with high-fat-fed intervention, accompanied by protection against insulin resistance (or in other words, enhanced insulin sensitivity). Although body weight tended to be lower in *Cdkal1* ^−/−^ mice on a standard diet throughout the follow-up period (Figure 2A), an inter-strain difference in %intraabdominal and %subcutaneous fat and lipid accumulation in the liver and muscle became most prominent after 8 weeks on a high-fat diet (Figure 2C and Figure S3A-S3C). After 16 weeks on a high-fat diet, when the inter-strain difference in %intraabdominal and %subcutaneous fat was no longer noticeable, insulin sensitivity assessed by a homeostasis model assessment of insulin resistance (HOMA-IR) was still significantly better in *Cdkal1* ^−/−^ mice (*P*<0.05, *n* = 5 for each strain) (Figure 2C). This enhanced insulin sensitivity is likely to surpass the potential impairment of insulin secretion and to result in a reduced impairment of glucose disposal during OGTT in *Cdkal1* ^−/−^ mice as compared to wild-type littermates in the early phase (Figure S4E, S4F and S4J).

To discuss a hypothetical molecular mechanism underlying the metabolic phenotypes of *Cdkal1* ^−/−^ mice, we performed microarray gene expression analysis on the muscle, which we chose as a primary target tissue because of the abundant expression of *Cdkal1* (Figure 1B) and its physiological importance in energy expenditure. It has turned out that a relatively small proportion of genes are differentially expressed in the muscle between *Cdkal1* ^+/+^and *Cdkal1* ^−/−^ mice at a fair cut-off level; e.g., 32 probesets were detectable by a filtering condition of |fold change|>1.5 and *P*<0.05 (Table S3), where we could not find strong evidence of enriched functionally-related genes. Among the 32 probesets, *Scd1* is remarkable in that *Scd1*-deficient mice show the metabolic phenotypes in accordance with those observed in *Cdkal1* ^−/−^ mice; e.g., decreased susceptibility to diet-induced obesity, decreased plasma insulin level, and improved glucose tolerance [32]. *Scd1* in adipose tissue is assumed to play a key role in the development of obesity [33]. We therefore validated by quantitative reverse-transcription PCR a significant reduction in *Scd1* mRNA expression in the muscle and WAT of *Cdkal1* ^−/−^ mice at an early stage but not at a later stage of high-fat feeding (Figures 5A and 5B). In *Cdkal1* transgenic mice, *Scd1* mRNA expression tended to be elevated in a reciprocal manner (Figure S1). Moreover, while a previous study [33] showed that Klf15 could suppress the expression of *Scd1* in adipose tissues, we have not obtained the results supporting it, considering the direction of *Klf15* expression changes in *Cdkal1* ^−/−^ mice and *Cdkal1* transgenic mice (Figure S6). Taken together, our data indicate that *Scd1* might function as a critical mediator of the altered metabolism in *Cdkal1* ^−/−^ mice. Further investigation is warranted to elucidate the network triggered by *Cdkal1* deletion, including the potential mechanisms beneath the insulin-independent glucose uptake (Figure S5).

In terms of genetic correlation between heritable quantitative traits, body weight, BMI, and waist circumference (all of which could represent intraabdominal fat) were found to cluster near serum insulin levels by a hierarchical clustering method, suggesting the presence of an etiological relationship between the traits [34]. This supports the bidirectional genetic effects of *Cdkal1–*reduced adiposity and increased susceptibility to type 2 diabetes. The complex interplay between insulin secretion, insulin sensitivity, and dietary habits in relation to glucose metabolism led us to hypothesize that type 2 diabetes risk loci including *CDKAL1* could exert some protective effects on fat accumulation, which was represented by BMI in the present study. As genetic effects of *Cdkal1* deletion on body weight reduction and protection against diet-induced obesity appeared to be rather modest in mice, we should be careful in extrapolating the findings in mice to humans. We identified a nominally significant (*P*<0.05) association between *CDKAL1* rs4712523 and BMI in 2 Japanese general populations; the effect size (in β) was almost comparable to that at *MC4R* rs12970134, one of the most robustly confirmed BMI-associated loci, while the direction of BMI association was reversed (β = −3.38 and 3.08 for *CDKAL1* and *MC4R*, respectively; Table S4). Among the loci thus tested, the largest effect size for BMI reduction was found at *TCF7L2*, where the OR for type 2 diabetes was also greatest in Japanese. In agreement with these findings, several previous studies in European populations have reported that the risk variant(s) of *TCF7L2* can be associated with lower BMI [35]–[37], whereas a few studies (involving<5,000 participants) have not detected significant association between measures of fatness and genetic variants of type 2 diabetes risk loci except *FTO* and *MC4R*; here, the risk variants of these 2 loci increase both type 2 diabetes susceptibility and BMI [38], [39] (Figure 6A). Large-scale studies of general populations are needed in multiple ethnic groups to gain more insight.

Another issue of note is the adiposity (or fat accumulation)-related heterogeneity in patterns of type 2 diabetes susceptibility. As a whole, there appears to be a tendency of inverse correlation between the OR for type 2 diabetes at *CDKAL1* and BMI (Figure 7). Along this line, a few previous studies in Europeans have shown that some genetic variants acting on insulin secretion (e.g., *TCF7L2*) have a greater impact on type 2 diabetes in non-obese subjects than in obese subjects [39], [40]. Dietary habits (i.e., non-genetic or environmental factors) and obesity susceptibility can collectively and interactively exert influences on BMI (or degree of obesity). In *Cdkal1* ^−/−^ mice, reduced fat accumulation is evident at the early stage but becomes inconspicuous after a certain period of high-fat feeding. Accordingly, it is possible that excess dietary fat intake, e.g., the Western diet, results in the attenuation of the genetic effect of *Cdkal1* on reduced fat accumulation (or BMI) within the population. Type 2 diabetes risk conferred by *CDKAL1* variant(s) can be appreciably modified by BMI (Figure 7) as a consequence of a gene–environment (or diet) interaction, in relation to ethnic-group-specific dietary habits, e.g., a Western diet vs. an East Asian diet.

It has been shown that a high fat–fed C57BL/6J mouse model is suitable for studies on islet dysfunction in combination with insulin resistance [41]–[43]. Compensatory adaptations of insulin secretion for insulin resistance have been reported to change over time in the high fat–fed mouse model during long-term (∼40 weeks after initiation of high-fat diet) studies [42]. That is, while there was no significant compensatory increase in the early insulin response to glucose in the high-fat diet after 4 weeks, the compensation (although insufficient for the total requirement) appeared after 12 weeks and persisted throughout the study period [42]. Thus, considering the coexistent, enhanced insulin sensitivity in addition to the assumed compensation in *Cdkal1* ^−/−^ mice, it is reasonable that long-term high fat–fed intervention, i.e., 20 weeks of high-fat feeding in the present study, was required to demonstrate the exaggerated glucose intolerance by OGTT, which could result from the eventual imbalance of compensatory mechanisms.

There are several limitations in the present study. Above all, detailed mechanisms linking a global *Cdkal1*-null mutation to fluctuations in fat accumulation remain to be defined. One plausible explanation is that the decreased food intake, resulting from the suppression of appetite, can drive the metabolic changes observed in *Cdkal1* ^−/−^ mice. However, the decreased food intake alone cannot account for fat and body weight increases in a later stage of high-fat feeding. It is therefore possible that the effect on fat accumulation is partly independent of the food intake, although a pair feeding experiment is required to verify this. Also, further investigations of the network involving *Scd1* are warranted to gain mechanistic insights into the altered insulin sensitivity in *Cdkal1* ^−/−^ mice.

In summary, the present data show that *Cdkal1* gene deletion is accompanied by modestly impaired insulin secretion and longitudinal fluctuations in insulin sensitivity during high-fat feeding in mice. As observed in the early phase, the compensatory mechanisms are appreciably modified by diet. In this context, data presented from two Japanese populations support the opposing effects of *CDKAL1* variants on BMI and type 2 diabetes.

*Note added in proof:* Since this manuscript was submitted, two BMI meta-analyses in East Asians were published [44], [45]. In these studies, a highly significant (*P*<1×10^−10^) association was detected for *CDKAL1*, in accordance with the present study in Japanese.

## Supporting Information

Figure S1 Increased body weight in *Cdkal1* transgenic mice. **(A)** Body weight curves from WT (open circle, *n* = 5) and *Cdkal1* transgenic (TG) mice (solid triangle, *n* = 4) fed on standard diet. *P*<0.001; F (1, 25) = 5.4 by repeated measure ANOVA. **(B)** Plasma concentrations of glucose (upper panel) and insulin (lower panel) in WT (open bar, *n* = 6) and TG mice (gray bar, *n* = 5), which were measured in the non-fasting state after 8-weeks of high-fat feeding. **(C)** Western blot analysis of Cdkal1 in the islet, brain, and liver isolated from KO, WT, and TG mice. The arrows and asterisks indicate Cdkal1 proteins and nonspecific bands, respectively. **(D)** Quantification of *Scd1* mRNA in skeletal muscle, WAT, BAT, and liver of WT (open bar, *n* = 4) and TG mice (gray bar, *n* = 4) fed on a high-fat diet for 10 weeks. The mRNA expression of *Scd1* was normalized to that of β-actin. The normalized data for TG mice are expressed relative to those for WT littermates. **P<0.01 by unpaired *t* test.
(PDF)

Figure S2 Assessment of spontaneous locomotor activity in WT (*n* = 4) and KO (*n* = 4) mice. No significant differences were observed between the two strains. **(A)** Total locomotor activity monitored in the open field for each 10-minute slot. **(B)** Total locomotor activity monitored in the open field for 60 min. Data are presented as mean ± SEM. N.S., not significant.
(PDF)

Figure S3 High fat feeding-induced lipid accumulation in the liver. Representative liver stained with hematoxylin and eosin **(A)**, and liver **(B)** and muscle **(C)** triglyceride content in wildtype littermates (open bar) and *Cdkal1* knockout mice (solid bar) fed on a high fat diet (HFD). [WT (*n* = 6–12) vs. KO (*n* = 5–9)]. *P<0.05, **P<0.01. Scale Bar = 50 µm.
(PDF)

Figure S4 Glucose tolerance and insulin sensitivity in *Cdkal1* knockout (KO) mice. Oral glucose tolerance tests (OGTT, 2 g/kg glucose; **A, E**) and insulin tolerance tests (ITT, 0.75 IU/kg insulin; **C, G**) in wild-type littermates (WT) and *Cdkal1* KO mice. In mice on a standard diet (at 12 and 20 weeks of age), (**A**) glucose tolerance was assessed by OGTT [WT (*n* = 12) vs. KO (*n* = 21)]; (**B**) the areas under the curve were assessed for blood glucose levels (AUC_glucose_) with inter-strain and inter-age-group comparison; (**C**) insulin sensitivity was assessed by ITT [WT (*n* = 10) vs. KO (*n* = 9)]; and (**D**) the areas under the curve were assessed for insulin levels in perfusate (AUC_insulin_) with the trapezoidal rule of suprabasal values. The corresponding results are shown in (**E**) to (**H**) for mice on a high fat diet (after 4 and 8 weeks of dietary intervention, 12 and 16 weeks of age) [WT (*n* = 11) vs. KO (*n* = 9) for OGTT; and WT (*n* = 11) vs. KO (*n* = 12) for ITT]. In (**B)**, (**D**), (**F**), and (**H**), open and solid bars are for WT and KO mice, respectively. Insulin levels during OGTT (0–30 min) are shown for mice on a standard diet at 12 weeks of age (**I**) and mice on a high fat diet after 8 weeks of dietary intervention (**J**); *P*<0.01, F (1, 2) = 6.6 by repeated measure ANOVA. *P<0.05, **P<0.01 by *t* test.
(PDF)

Figure S5 Examination of insulin-dependent Akt activation in *Cdkal1* knockout (KO) mice-derived tissues. Western blot analysis of total- and phospho-Akt (pAkt) in liver, skeletal muscle and white adipose tissue (WAT). At the fasted state, WT and KO mice fed on a high-fat diet for 4 weeks were administered with insulin via the inferior vena cava. The livers were removed at 2 min, the hind limb muscles and white adipose tissues (WAT) removed at 5 min after injection. The lysates were immunoblotted with total- and pAkt antibody, respectively. Experiments were performed in duplicate and similar results obtained. Relative intensity of phospho-Akt level is calculated with normalization to total-Akt content (lower panel). Data for WT (open bar) and KO (solid bar) mice are presented as mean ± SEM.
(PDF)

Figure S6 *Klf15* expression in white adipose tissues of *Cdkal1* transgenic mice. *Klf15* mRNA expression was increased at the early stage of high fat diet. *Klf15* mRNA was quantified in skeletal muscle, WAT, BAT and liver of WT (open bar, *n* = 4), KO (solid bar, *n* = 4) and TG mice (gray bar, *n* = 4) fed on a high-fat diet for 10 weeks. The mRNA expression of *Klf15* was normalized to that of β-actin. The normalized data for KO and TG mice are expressed relative to those for WT littermates. *P<0.05 by unpaired *t* test.
(PDF)

Table S1 Diet compositions.
(XLS)

Table S2 A list of primers used for qRT-PCR analysis.
(XLS)

Table S3 A list of gene showing mRNA expression changes (fold≥|1.25|, *P*<0.05) in the skeletal muscle between *Cdkal1* ^−/−^ and wild-type mice.
(XLS)

Table S4 Cohort-wise BMI association of SNPs genotyped in two general Japanese populations, restricted to non-diabetics.
(XLS)

Table S5 Meta-analysis of type 2 diabetes association at the *CDKAL1* locus.
(XLS)

Text S1 Supporting information on animals and human subjects.
(PDF)
