# Supplementary figures and images for: Deletion of CDKAL1 Affects High-Fat Diet–Induced Fat Accumulation and Glucose-Stimulated Insulin Secretion in Mice, Indicating Relevance to Diabetes
Source: PLoS One. 2012 Nov 16;7(11):e49055. doi: 10.1371/journal.pone.0049055 (PMC3500257; doi:10.1371/journal.pone.0049055)

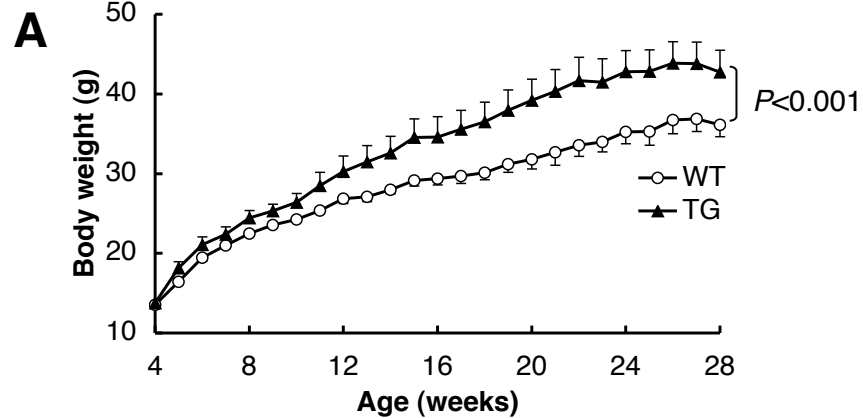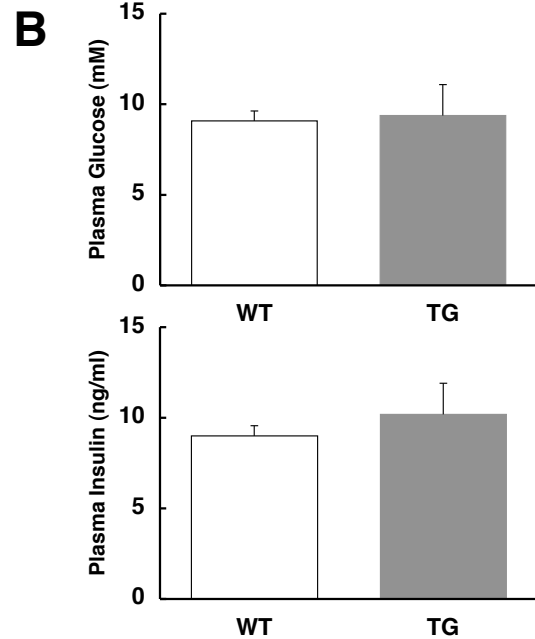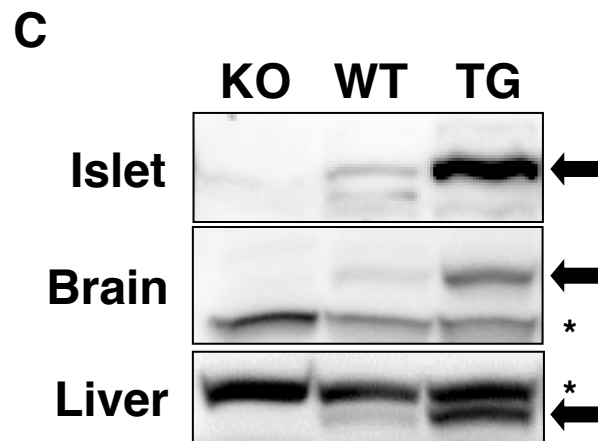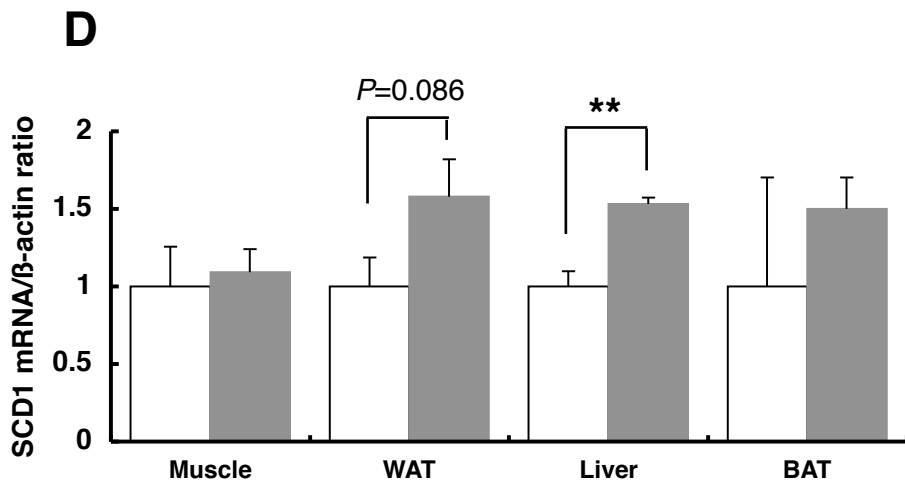

Supplement: Figure S1 — Increased body weight in Cdkal1 transgenic mice. (A) Body weight curves from WT (open circle, n = 5) and Cdkal1 transgenic (TG) mice (solid triangle, n = 4) fed on standard diet. P<0.001; F (1, 25) = 5.4 by repeated measure ANOVA. (B) Plasma concentrations of glucose (upper panel) and insulin (lower panel) in WT (open bar, n = 6) and TG mice (gray bar, n = 5), which were measured in the non-fasting state after 8-weeks of high-fat feeding. (C) Western blot analysis of Cdkal1 in the islet, brain, and liver isolated from KO, WT, and TG mice. The arrows and asterisks indicate Cdkal1 proteins and nonspecific bands, respectively. (D) Quantification of Scd1 mRNA in skeletal muscle, WAT, BAT, and liver of WT (open bar, n = 4) and TG mice (gray bar, n = 4) fed on a high-fat diet for 10 weeks. The mRNA expression of Scd1 was normalized to that of β-actin. The normalized data for TG mice are expressed relative to those for WT littermates. **P<0.01 by unpaired t test. (PDF) [file pone.0049055.s001.pdf]

**A**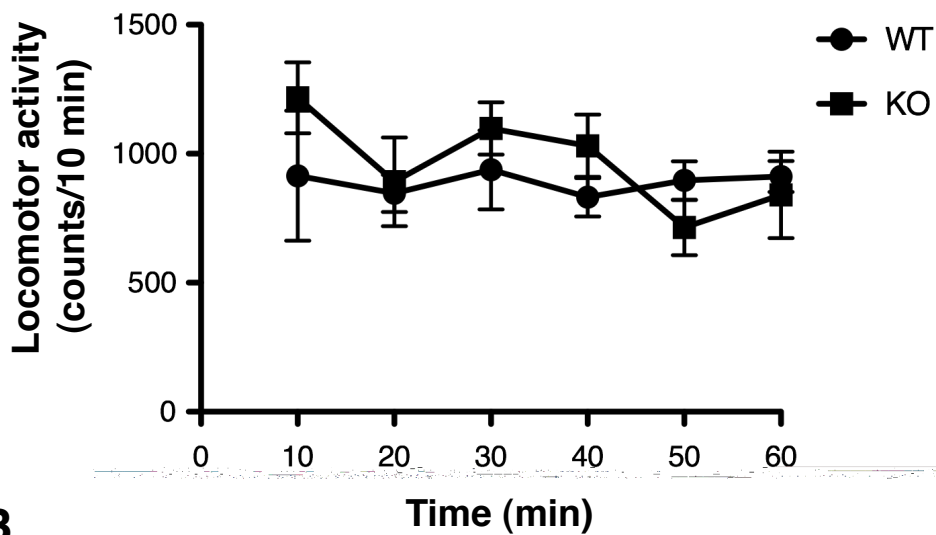**B**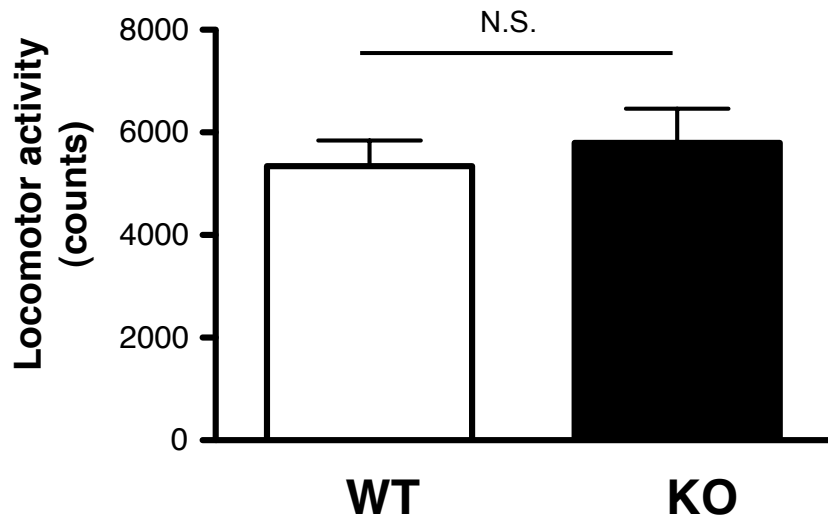

Supplement: Figure S2 — Assessment of spontaneous locomotor activity in WT (n = 4) and KO (n = 4) mice. No significant differences were observed between the two strains. (A) Total locomotor activity monitored in the open field for each 10-minute slot. (B) Total locomotor activity monitored in the open field for 60 min. Data are presented as mean ± SEM. N.S., not significant. (PDF) [file pone.0049055.s002.pdf]

**A***Cdkal1*<sup>+/+</sup>*Cdkal1*<sup>-/-</sup>4wks  
of HFD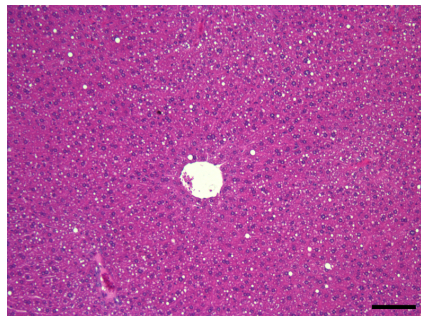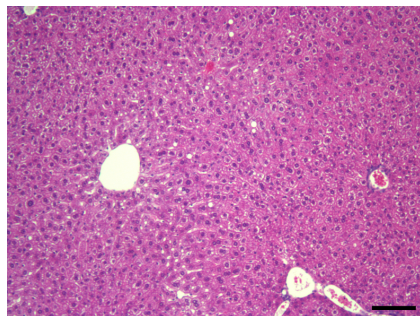8wks  
of HFD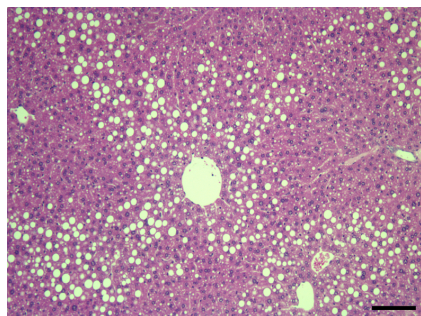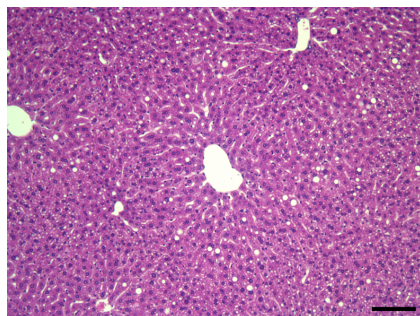20wks  
of HFD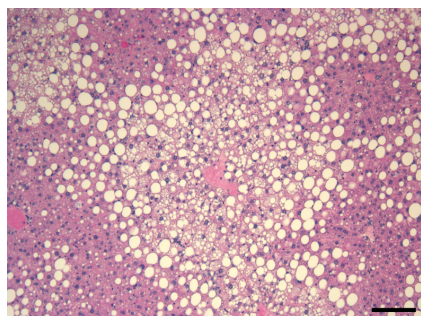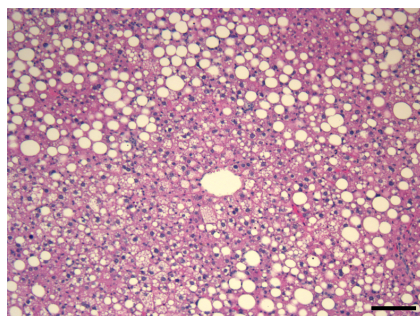**B**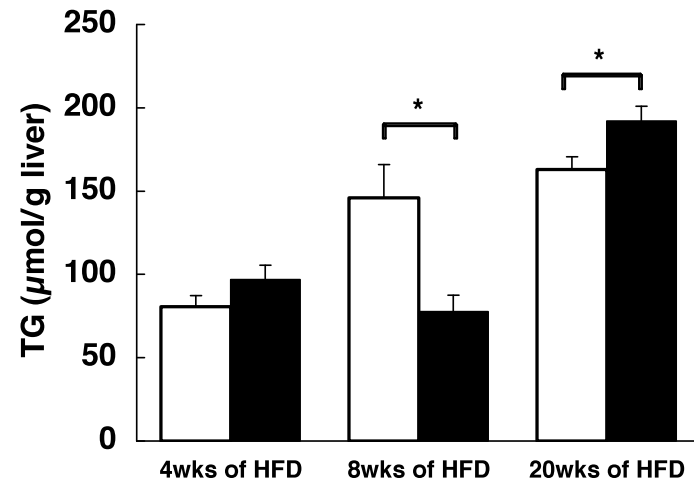**C**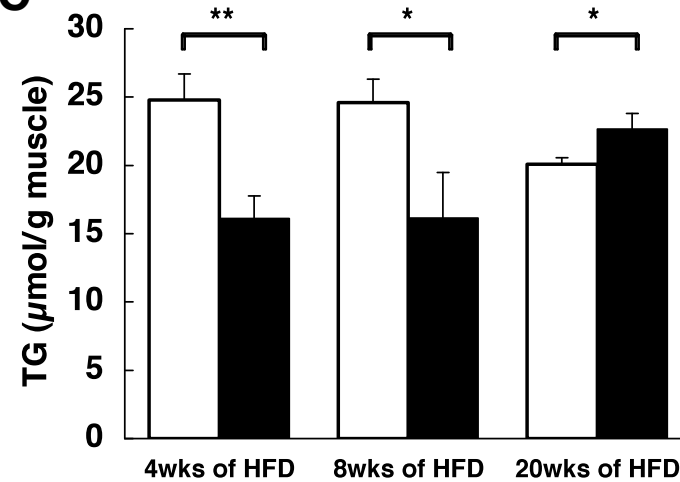

Supplement: Figure S3 — High fat feeding-induced lipid accumulation in the liver. Representative liver stained with hematoxylin and eosin (A), and liver (B) and muscle (C) triglyceride content in wildtype littermates (open bar) and Cdkal1 knockout mice (solid bar) fed on a high fat diet (HFD). [WT (n = 6–12) vs. KO (n = 5–9)]. *P<0.05, **P<0.01. Scale Bar = 50 µm. (PDF) [file pone.0049055.s003.pdf]

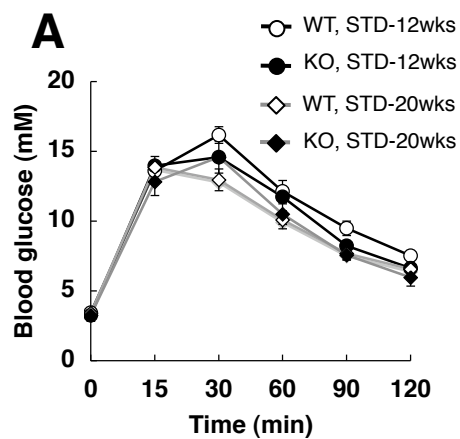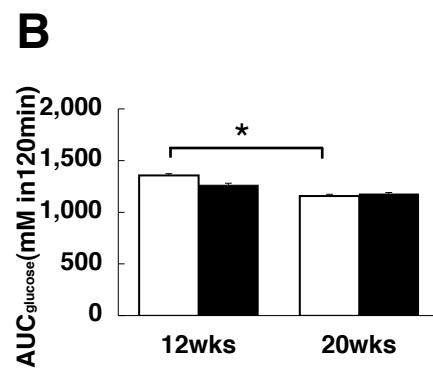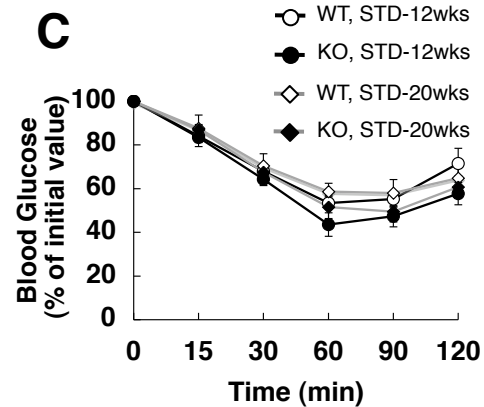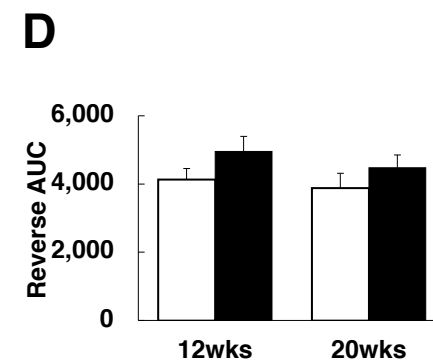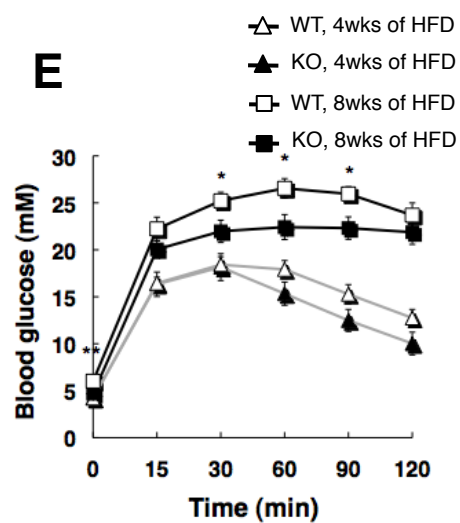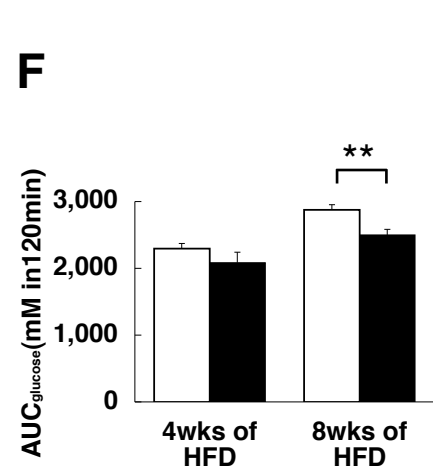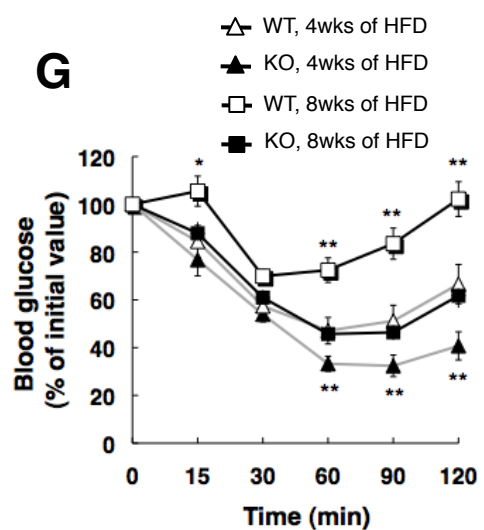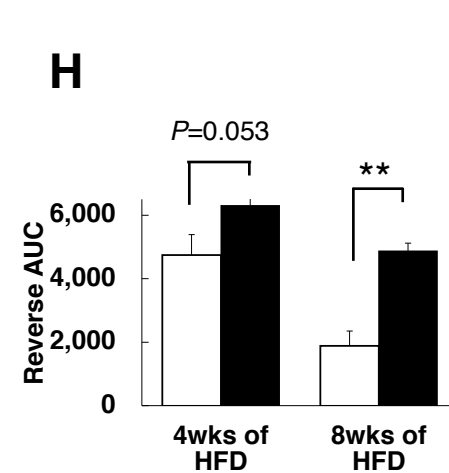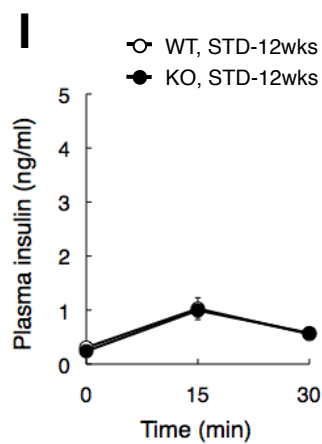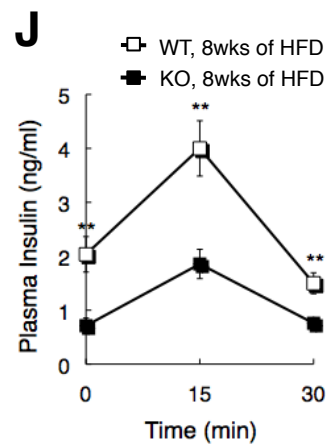

Supplement: Figure S4 — Glucose tolerance and insulin sensitivity in Cdkal1 knockout (KO) mice. Oral glucose tolerance tests (OGTT, 2 g/kg glucose; A, E) and insulin tolerance tests (ITT, 0.75 IU/kg insulin; C, G) in wild-type littermates (WT) and Cdkal1 KO mice. In mice on a standard diet (at 12 and 20 weeks of age), (A) glucose tolerance was assessed by OGTT [WT (n = 12) vs. KO (n = 21)]; (B) the areas under the curve were assessed for blood glucose levels (AUCglucose) with inter-strain and inter-age-group comparison; (C) insulin sensitivity was assessed by ITT [WT (n = 10) vs. KO (n = 9)]; and (D) the areas under the curve were assessed for insulin levels in perfusate (AUCinsulin) with the trapezoidal rule of suprabasal values. The corresponding results are shown in (E) to (H) for mice on a high fat diet (after 4 and 8 weeks of dietary intervention, 12 and 16 weeks of age) [WT (n = 11) vs. KO (n = 9) for OGTT; and WT (n = 11) vs. KO (n = 12) for ITT]. In (B), (D), (F), and (H), open and solid bars are for WT and KO mice, respectively. Insulin levels during OGTT (0–30 min) are shown for mice on a standard diet at 12 weeks of age (I) and mice on a high fat diet after 8 weeks of dietary intervention (J); P<0.01, F (1, 2) = 6.6 by repeated measure ANOVA. *P<0.05, **P<0.01 by t test. (PDF) [file pone.0049055.s004.pdf]

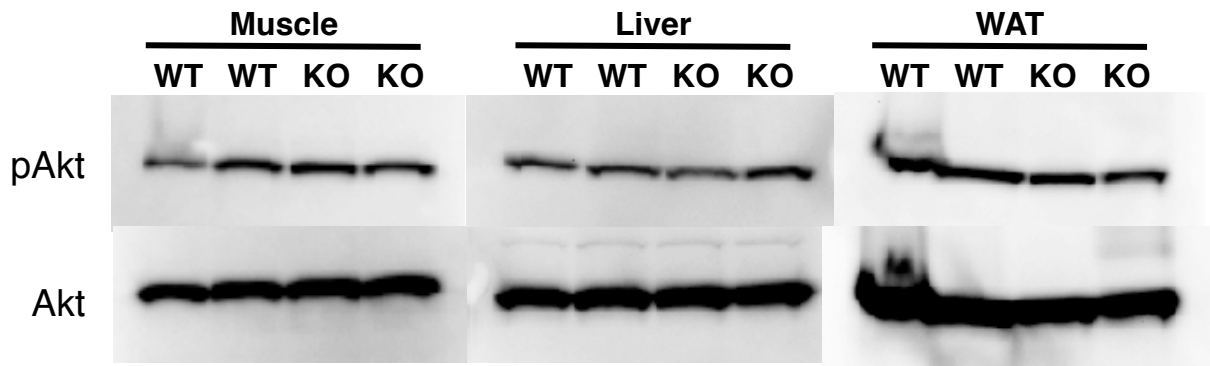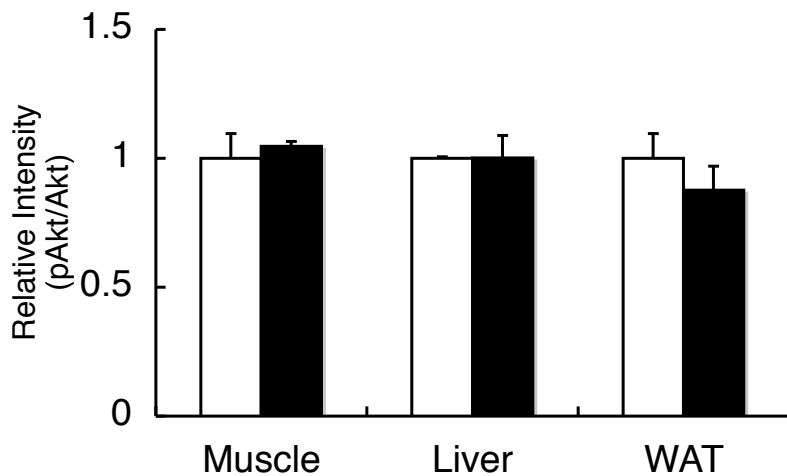

Supplement: Figure S5 — Examination of insulin-dependent Akt activation in Cdkal1 knockout (KO) mice-derived tissues. Western blot analysis of total- and phospho-Akt (pAkt) in liver, skeletal muscle and white adipose tissue (WAT). At the fasted state, WT and KO mice fed on a high-fat diet for 4 weeks were administered with insulin via the inferior vena cava. The livers were removed at 2 min, the hind limb muscles and white adipose tissues (WAT) removed at 5 min after injection. The lysates were immunoblotted with total- and pAkt antibody, respectively. Experiments were performed in duplicate and similar results obtained. Relative intensity of phospho-Akt level is calculated with normalization to total-Akt content (lower panel). Data for WT (open bar) and KO (solid bar) mice are presented as mean ± SEM. (PDF) [file pone.0049055.s005.pdf]

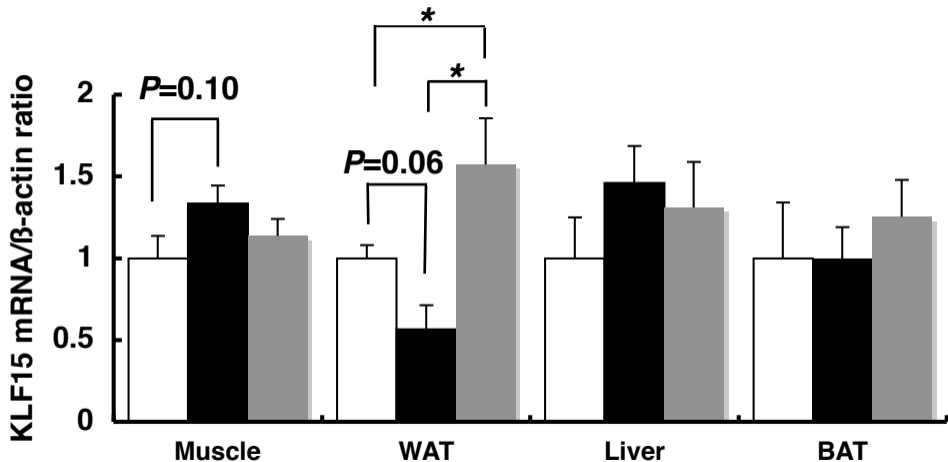

Supplement: Figure S6 — Klf15 expression in white adipose tissues of Cdkal1 transgenic mice. Klf15 mRNA expression was increased at the early stage of high fat diet. Klf15 mRNA was quantified in skeletal muscle, WAT, BAT and liver of WT (open bar, n = 4), KO (solid bar, n = 4) and TG mice (gray bar, n = 4) fed on a high-fat diet for 10 weeks. The mRNA expression of Klf15 was normalized to that of β-actin. The normalized data for KO and TG mice are expressed relative to those for WT littermates. *P<0.05 by unpaired t test. (PDF) [file pone.0049055.s006.pdf]
