# Supplementary material for: Deletion of CDKAL1 Affects High-Fat Diet–Induced Fat Accumulation and Glucose-Stimulated Insulin Secretion in Mice, Indicating Relevance to Diabetes
Source: PLoS One. 2012 Nov 16;7(11):e49055. doi: 10.1371/journal.pone.0049055 (PMC3500257; doi:10.1371/journal.pone.0049055)
Supplement: Text S1 — Supporting information on animals and human subjects. (PDF) [file pone.0049055.s012.pdf]

Animals

1) Generation of *Cdkal1* transgenic mice

A full-length cDNA of mouse *Cdkal1* was inserted into the pCXN expression vector [47], which contained the cytomegalovirus immediate-early enhancer, chicken β-actin promoter, and rabbit β-globin polyadenylation signal (a ubiquitous, CAG promoter). We generated transgenic mice using standard procedures [48]. The linearized transgene was microinjected at the concentration of 2 ng/μl into the pronuclei of the fertilized eggs of C57BL/6NCr mice (SLC Inc., Hamamatsu, Japan). The presence of the transgene in founder mice and their progeny was detected by PCR amplification of genomic DNA from tail biopsy, using a pair of primers; 5'-CAGAGGCGGAATACCCAAA-3' and 5'-ATTATGTGAGCAGCCCCATGTTC -3'. Two independent transgenic lines were established.

Human Subjects

1) Study samples for continuous traits

Amagasaki panel: To investigate lifestyle factors and genetic susceptibility to cardiovascular diseases and risk factor traits, we enrolled individuals who sought medical assessment from September 2002 to August 2003 at the Amagasaki Health Medical Foundation, as described elsewhere [20]. The participants were included if they were over 18 years of age and had full clinical examination data, along with a completed questionnaire on their lifestyle. On the basis of these inclusion criteria, 5,695 individuals (3,405 males and 2,290 females) were initially enrolled in this prospective cohort study, known as the Amagasaki Study. A subgroup of 4,813 subjects, whose blood samples were collected after ≥6 h fast without glucose lowering medication, were used for BMI-associated SNP-trait association analysis.

Fukuoka panel: The study subjects were participants in the baseline survey of the Kyushu University Fukuoka Cohort Study, which was designed to investigate lifestyle

factors and genetic susceptibility of the so-called lifestyle-related diseases such as cardiovascular diseases, cancer, and diabetes mellitus. Eligible persons were residents of the East Ward of Fukuoka City aged 50 to 74 years. Some areas in the Ward were excluded because of potential emigration, sparse population, and remote distance. Of 53,927 persons who were contacted by mail, a total of 12,959 participated in the baseline survey during the period from February 2004 to August 2007. Brief description of the methods in the baseline survey is available elsewhere [21]. Excluding 8 for withdrawal, one for duplicate participation, and one for mental incompetence, the cohort comprised 12,949 persons. Of them, 12,629 gave informed consent to genetic analysis. For DNA extraction, 6 ml of venous blood was drawn into a vacuum tube containing EDTA-K2. Within at most 8 hours of blood sampling, 2 ml of plasma was taken after centrifugation, and the remaining blood sample added with 1 ml PBS was stored at –80 °C until DNA extraction. DNA was extracted from the peripheral leukocyte fraction using automatic nucleic acid isolation system NA-3000 (Kurabo, Tokyo, Japan) and stored at –80 °C until genotyping. A total of 12,569 subjects completed the questionnaire and also provided DNA for genotyping of SNPs.

Since blood was drawn not strictly on the condition of overnight fast, HbA1c was measured among all participants in the Kyushu University Fukuoka Cohort Study to evaluate the impaired glucose metabolism.

2) Study samples for T2D case-control study

Cases and unaffected controls for T2D association study were all derived from the participants in the GWA study of Japanese previously reported [5]. Briefly, T2D cases included 1,629 subjects who were enrolled according to the 1999 WHO criteria from the clinical practice or the annual medical checkup of university hospitals, medical institutions, and general practitioners that constitute the Study Group of the National Institute of Biomedical Innovation Organization (NIBIO) GWA, plus 4,000 T2D

subjects derived from the registered samples in the Biobank Japan (http://biobankjp.org/). Also, 1,517 unaffected controls were included according to the following criteria: (1) no past history of urinary glucose or glucose intolerance; (2) HbA1c, <5.6% or a normal glucose (75g) tolerance test; and (3) age at examination, ≥55 years. Moreover, from the population-based subjects in the Fukuoka panel (n = 12,569), diabetic subjects (n = 740) and unaffected controls (n = 4,889) were selected for case-control analysis. Here (in the Fukuoka panel), diabetes was defined as HbA1c ≥7.0 or under treatment of diabetes; the controls were chosen as non-diabetic subjects who met the following conditions: age, ≥55 years; HbA1c, ≤5.0%; no previous and/or current treatment for diabetes; and absence of renal failure (serum creatinine, <3.0 mg/dl), as previously described [21]. In total, 6,369 cases and 6,406 controls were used for the T2D case-control study in the Japanese.

Diabetic subjects from the BioBank Japan (http://biobankjp.org/) were categorized into three BMI groups, <25, 25–30 and ≥30, for which we used representative values of 22.5, 27.5 and 32.5 in the adjustment for BMI.

References

References up to number 46 are listed in the main text.

47. Niwa H, Yamamura K, Miyazaki J (1991) Efficient selection for high-expression transfectants with a novel eukaryotic vector. Gene 180: 193–200.

48. Okamura T, Miyoshi I, Takahashi K, Mototani Y, Ishigaki S, et al. (2003) Bilateral congenital cataracts result from a gain-of-function mutation in the gene for aquaporin-0 in mice. Genomics 81: 361-368.
